# Supplementary material for: Monophasic Variant of Salmonella Typhimurium 4,[5],12:i:- (ACSSuGmTmpSxt Type) Outbreak in Central Italy Linked to the Consumption of a Roasted Pork Product (Porchetta)
Source: Microorganisms. 2023 Oct 15;11(10):2567. doi: 10.3390/microorganisms11102567 (PMC10609469; doi:10.3390/microorganisms11102567)
Supplement: Supplementary file 1 [file microorganisms-11-02567-s001.zip › microorganisms-2657111-supplementary.pdf]

Table S1: Total number (n=25) of food (n=2) and environmental (n=23) samples at RS(A) (n=10), RS(B) (n=6) and FPP (n=9); *(FCS): surface in contact with food, **(NFCS): surface not in contact with food.

Sampling dates	Sampling place	Sample detail	Origin sampling	Salmonella result
17 August	RS(A)	Sponge swab on unsanitized surface (NFCS)**	Anteroom access door handle	Negative
17 August	RS(A)	Sponge swab on unsanitized surface (FCS)*	"Porchetta" knife	Negative
17 August	RS(A)	Sponge swab on unsanitized surface (FCS)*	"Porchetta" picking scoop	Negative
17 August	RS(A)	Sponge swab on unsanitized surface (FCS)*	Teflon chopping board for supporting and cutting "porchetta"	Positive
17 August	RS(A)	Sponge swab on unsanitized surface (FCS)*	Teflon cutting board from the butcher's	Negative
17 August	RS(A)	Sponge swab on unsanitized surface (FCS)*	Teflon worktop in butcher's	Negative
17 August	RS(A)	Sponge swab on unsanitized surface (FCS)*	Teflon cutting board for white meat counter	Positive
17 August	RS(A)	Sponge swab on unsanitized surface (NFCS)*	Fresh meat area scale keyboard	Negative
17 August	RS(A)	Sponge swab on unsanitized surface (FCS)*	Fresh meat slicer	Negative
17 August	RS(A)	Sponge swab on unsanitized surface (FCS)*	Teflon cutting board behind the counter	Negative
23 August	RS(B)	Ready-to-eat food	"Porchetta"	Positive
23 August	RS(B)	Sponge swab on unsanitized surface (FCS)*	Wooden chopping board for "porchetta"	Positive
23 August	RS(B)	Sponge swab on unsanitized surface (FCS)*	"Porchetta" knife	Positive
23 August	RS(B)	Sponge swab on unsanitized surface (FCS)*	"Porchetta" spatula	Negative
23 August	RS(B)	Sponge swab on unsanitized surface (FCS)*	Teflon cutting board to the right of the wooden cutting board	Negative
23 August	RS(B)	Sponge swab on unsanitized surface (FCS)*	Steel table to the left of the wooden chopping board	Negative
24 August	FPP	Ready-to-eat food	"Porchetta"	Negative
24 August	FPP	Sponge swab on unsanitized surface (FCS)*	Knife	Negative
24 August	FPP	Sponge swab on unsanitized surface (FCS)*	Refrigerator grill	Negative

24 August	FPP	Sponge swab on unsanitized surface (NFCS)*	Refrigerator wall	Negative
24 August	FPP	Sponge swab on unsanitized surface (FCS)*	Transporting board for cooked “porchetta”	Positive
24 August	FPP	Sponge swab on unsanitized surface (NFCS)**	Refrigerator bottom	Negative
24 August	FPP	Sponge swab on unsanitized surface (NFCS)**	Refrigerator handle	Negative
24 August	FPP	Sponge swab on sanitized surface (FCS)*	Raw “porchetta” manipolating table	Negative
24 August	FPP	Sponge swab on sanitized surface (FCS)*	Knife	Negative
